# Physician Experiences with Communicating Organ Donation with the Relatives: A Dutch Nationwide Evaluation on Factors that Influence Consent Rates

**DOI:** 10.1007/s12028-019-00678-8

**Published:** 2019-02-14

**Authors:** Marloes Witjes, P. Edwin Vorstius Kruijff, Bernadette J. J. M. Haase-Kromwijk, Johannes G. van der Hoeven, Nichon E. Jansen, Wilson F. Abdo

**Affiliations:** 1 Department of Intensive Care Medicine, Radboud University Medical Center, P.O. Box 9101, Internal Post 710, 6500 HB Nijmegen, The Netherlands; 2 Dutch Transplant Foundation, Leiden, The Netherlands; 3 Department of Quality and Safety, Amphia Hospital, Breda, The Netherlands

**Keywords:** Tissue and organ procurement, Health communication, Family

## Abstract

**Background:**

The aim of this nationwide observational study is to identify modifiable factors in communication about organ donation that influence family consent rates.

**Methods:**

Thirty-two intensivists specialized in organ donation systematically evaluated all consecutive organ donation requests with physicians in the Netherlands between January 2013 and June 2016, using a standardized questionnaire.

**Results:**

Out of 2528 consecutive donation requests, 2095 (83%) were evaluated with physicians. The questionnaires of patients registered with consent or objection in the national donor registry were excluded from analysis. Only those questionnaires, in which the family had to make a decision about donation, were analyzed (*n* = 1322). Independent predictors of consent included: requesting organ donation during the conversation about futility of treatment (OR 1.8; *p* = 0.004), understanding of the term ‘brain death’ by the family (OR 2.4; *p* = 0.002), and consulting a donation expert prior to the donation request (OR 3.4; *p* < 0.001).

**Conclusions:**

Our study showed that decoupling the organ donation conversation from the conversation about futility of treatment was associated with lower family consent rates. Comprehension of the concept of brain death by the family and consultation with a transplant coordinator before the organ donation request by the physician could positively influence consent rates.

**Electronic supplementary material:**

The online version of this article (10.1007/s12028-019-00678-8) contains supplementary material, which is available to authorized users.

## Introduction

In practically every country, organ donation is only allowed with the explicit consent of the potential donor or with consent to donation by the next of kin. The role of the family depends largely on the type of legal consent system used. The legal consent system used in the Netherlands, and many other countries such as the UK and USA, is an opting-in system. In such an opt-in system, people have to explicitly register their donation preferences. Lack of registrations is the major issue in countries with an opt-in system. For instance, in the Netherlands, 60% of the Dutch population of 18 years and older have not registered any choice. In these cases, the next of kin needs to make a decision at a very emotional moment, which leads to objection in 70% of the cases in 2017 [1, 2]. When patients have registered an explicit consent in the donor registry (DR), family consent rates are 87–94% in the Netherlands [2, 3]. The combined consent rate, including consent registrations, was 45% in 2017 [2].

The family refusal rate is one of the main bottlenecks in countries, irrespective of the legal consent system. The organ donation requests occur when the family is at a high emotional burden as their loved one is dying. If the potential organ donor has a registered donor preference, this helps guide the family in their decision. However, communication skills of the person requesting donation are also important. Understandably, many initiatives have focused on ‘how to communicate about donation with grieving relatives’ in the hope this increases family consent rates [4, 5]. Communication factors that seem to increase consent rates are: for example, the timing of the request [6], adequate information and understanding of brain death [7], making the request in a private setting [8], and using trained and experienced individuals to make the request [1].

### Initiatives in the Netherlands

In an effort to improve organ donation practices in the Netherlands, the Ministry of Health issued funding for a nationwide donation program which started in 2008 [9]. This program consisted of a set of initiatives that was based on recommendations of an advisory group to the Minister of Health. One of these initiatives was to allocate funding for ‘donation intensivists,’ which started in 2012. In total, 32 intensivists were appointed nationally to specialize in organ donation. Each donation intensivist was assigned to a specific region surrounding his or her main hospital. Their tasks were improving logistics and management of donation practices in their assigned region, strengthening collaboration within their donation network, analyzing data in their donation, providing education, and performing scientific research. The focus of the nationwide effort was not necessarily on changing the medical protocols and guidelines, but on improving the organ donation networks and logistics. In addition, intensivists were able to call the donation intensivist assigned to their region 24/7 if they had any medical questions or logistical issues surrounding a potential organ donor.

In addition, a nationwide ‘Communication about Donation’ (CaD) training was developed in 2007 for Dutch intensivists, other physicians, and nurses involved in organ donation requests, with the aim to increase donation consent rates. The training was modified and became obligatory for Dutch intensivists at the end of the year 2012. This training consists of an e-learning module and a 4-h practical training, including role-playing with actors. It focuses on communication techniques with the next of kin and how to guide them in the decision-making process for donation [10]. Since September 2007 (until December 2017), approximately 5300 physicians and nurses followed the CaD training.

### Study Aim

The aim of our nationwide observational study was to evaluate the effect of different modifiable factors in the decision-making process on the consent rate.

## Methods

One of the tasks of the donation intensivist is to evaluate all consecutive organ donation requests of physicians/intensivist, in their assigned region using a standardized questionnaire. The donation intensivist interviewed the physicians who approached the next of kin for the donation request and used the standardized questionnaire for these evaluations. In this nationwide study, we used the data of the questionnaires retrieved from January 2013 through June 2016. All potential organ donors and the occurrence of an organ donation request were registered, by a nationwide network of donation coordinators, in a national database. Once these patients were registered in the database, donation intensivists were directly informed about these potential donors and that a donation request had occurred. The donation intensivists then approached the physicians who performed the donation request for an evaluation per e-mail, in a face-to-face setting, or by telephone.

Requests for donation after brain death, as well as requests for donation after circulatory death, were evaluated as both pathways to donate are possible in the Netherlands. Evaluations were not performed when the potential donor was registered with ‘objection’ in the Dutch DR, as in these cases there would be no donation request. To prevent an overestimation of the consent rate, only the questionnaires of patients registered with ‘decision by next of kin/specific person’ and patients who were not registered, were analyzed as in these circumstances it is more difficult for families to make a decision, leading to a high refusal rate.

Donation intensivists, the Dutch Transplant Foundation, and donation and transplant coordinators in collaboration with the Dutch Society of Intensive Care, developed the standardized questionnaire (Additional file 1). The questions and answers were chosen as important areas for possible improvement that needed more data based on the literature and experience of the participants involved. The questionnaire consisted of multiple choice questions and had open fields to elaborate on answers if needed. The questionnaire consisted of 31 items describing the conditions, in which the family conversations took place. Items discussed were e.g.,: training level of the physician, consultation of a transplant coordinator, consultation of the DR, number of family members present during the donation conversation, understanding of the term ‘brain death’ (as judged by the requesting physician), whether or not a donation intensivist assisted, and decoupling of donation request from the conversation about futility of treatment. The questionnaire was first pilot-tested in one of the donation regions in the Netherlands before it was implemented nationally. Data included in this study did not include the pilot phase.

The conversation about futility of treatment is the explanation to the next of kin that further treatment is futile and, therefore, life-sustaining treatments will be withdrawn. In the Netherlands, it is the practice (as described in a nationwide protocol on organ donation that all hospitals have to follow) that the decision about futility of treatment is made before (and independently of) any decision on organ donation (including consultation of the national DR or consultation with the transplant coordinators). The brain death protocol will only be entered if, first, the decision of futility of treatment was made and, second, the family consented to heart beating organ donation. As such, the brain death protocol in the Netherlands is almost solely used for patients that donate their organs. This is different from countries where brain death is (primarily) used to determine futility of treatment.

The primary study outcome was the rate of family consent. The consent rates were compared by univariate analysis for all variables using two-tailed Pearson’s Chi-square test. When one of the groups was smaller than 50, the Fisher’s test (2 × 2 table) or Fisher-Freeman-Halton test (more than 2 × 2 table) was used. Variables with *p* < 0.20 were entered into a multiple binary logistic regression model to assess the independent predicting factors. Factors were added to the model in a forward step-wise fashion. The analyses were performed using SPSS (IBM), version 22.

Besides modifiable factors that might influence consent rate, the questionnaire also included descriptive factors. These were family-related factors that, to the opinion of the requesting physician, played a role in the decision-making process of the family, and general improvement points mentioned by physicians requesting donation (Additional file 1, questions 22 and 25). The answer options were predefined by professionals in the field and were direct examples from practice.

## Results

**Fig. 1:**
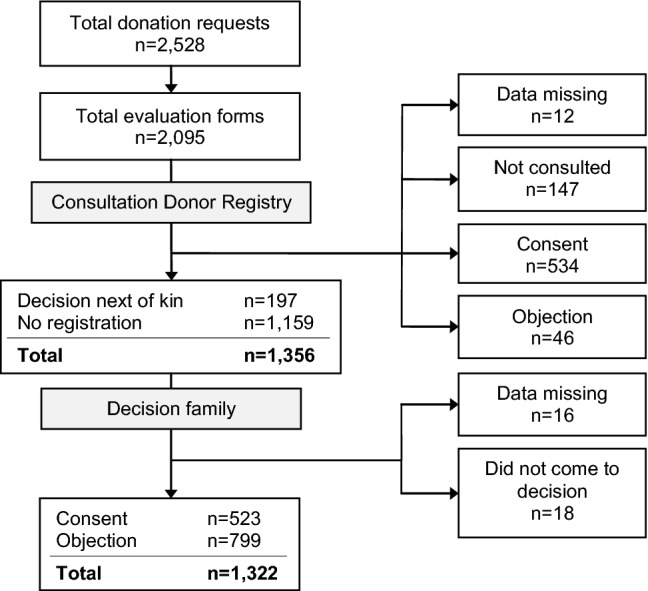
Flow chart showing the inclusion of evaluation forms

During the 3.5-year study period, there were 2528 donation requests nationally of which 2095 (83%) donation requests were evaluated by 32 donation intensivists using the questionnaire (Fig. 1). In total, evaluations took place in 89 hospitals in the Netherlands, which is 100% of all Dutch hospitals with an intensive care unit (ICU) (excluding cancer—and private hospitals where organ donation will not take place). In case, the potential donor was registered with consent in the DR (*n* = 534), the family approved to organ donation in 490 cases (91.8%) and objected in 34 (6.4%), while in 10 cases (1.9%) no decision was made or data on the decision were missing. An evaluation was performed in 46 cases while the patient had already registered an objection in the DR. However, in these cases, organ donation could never have occurred as the potential organ donor him- or herself objected, i.e., family could not have consented. Therefore, these 46 evaluations were excluded for further analysis. Cases were also excluded when the DR was not consulted, when the decision about donation was not made because of special circumstances (e.g., family too emotional or absent), when consent was registered in the DR, or when data on the decision were missing.

After applying these exclusion criteria, a total of 1322 questionnaires were analyzed (Fig. 1). In 194 (14.7%) cases, the potential donor was registered with ‘decision by next of kin/specific person’ in the DR and 1128 (85.3%) potential donors were not registered. Of the 1322 evaluations with the physicians, the donation intensivists performed 411 (31.1%) evaluations per e-mail, 510 (38.6%) in a face-to-face setting, 89 (6.7%) by telephone, 52 (3.9%) ‘other,’ and of 260 (19.7%) evaluations the data on how the evaluation was performed were missing. In the group ‘other,’ it was mostly mentioned that the donation intensivist used the questionnaire to evaluate a donation request he/she made him/herself. The median time between the donation request and evaluation with the physician who performed the donation request was 13.0 (IQR 3.0–34.0) days. For the multiple logistic regression, the outcomes of the question whether the family understood brain death were made dichotomous (completely versus partly/not).

Overall, 523 (39.6%) families consented and 799 (60.4%) families objected to donation. Table 1 shows the influence of different modifiable factors on the family consent rate. After a multiple logistic regression, independent predictors of consent to donation included consulting a transplant coordinator (OR 3.4; 95% CI 2.3–5.0; *p* < 0.001), complete understanding of the term ‘brain death’ by the family (OR 2.4; 95% CI 1.4–4.2; *p* = 0.001), explicitly asking if the family understood ‘brain death’ (OR 1.7; 95% CI 1.1–2.5; *p* = 0.010), and requesting organ donation during the conversation about futility of treatment (OR 1.8; 95% CI 1.2–2.6; *p* = 0.004). When all these four factors were present, the consent rate was 72.2% versus 15.8% when the four factors were not present (*p* < 0.001).

**Table 1:** Modifiable factors influencing the family consent rate (*n* = 1322)

Factors in donation process		Donation decision			*p* value
		Consent *n* (%)	Objection *n* (%)	Odds Ratio (95% CI)	
Was the transplantation coordinator consulted before the donation request?	Yes	286 (59.7)	193 (40.3)	4.088 (3.209–5.207)	< 0.001
	No	207 (26.6)	571 (73.4)	Ref.	
	Missing	30	35		
Did the physician requesting donation have assistance of a donation intensivist?^a^	Yes	106 (47.3)	118 (52.7)	1.426 (1.067–1.906)	0.016
	No	410 (38.6)	651 (61.4)	Ref.	
	Missing	7	30		
Did the physician requesting donation have any contact with the family during hospital admission?	Yes	428 (39.7)	651 (60.3)	1.033 (0.773–1.380)	0.825
	No	91 (38.8)	143 (61.1)	Ref.	
	Missing	4	5		
Did the physician requesting donation talk about donation with the family during hospitalization?	Yes	259 (41.2)	370 (58.8)	1.157 (0.926–1.444)	0.199
	No	256 (37.7)	423 (62.3)	Ref.	
	Missing	8	6		
How many family members were present during conversation about futility of treatment?	1–2 persons	106 (39.7)	161 (60.3)	Ref.	0.354^b^
	3–4 persons	220 (38.2)	356 (61.8)	0.939 (0.697–1.264)	
	5–6 persons	140 (43.8)	180 (56.3)	1.181 (0.850–1.642)	
	7 or more persons	41 (36.3)	72 (63.7)	0.865 (0.549–1.364)	
	Missing	16	30		
How many family members were present during organ donation request?	1–2 persons	104 (37.4)	174 (62.6)	Ref.	0.344^b^
	3–4 persons	232 (39.8)	351 (60.2)	1.106 (0.824–1.484)	
	5–6 persons	125 (43.6)	162 (56.4)	1.291 (0.922–1.808)	
	7 or more persons	39 (35.1)	72 (64.9)	0.906 (0.572–1.435	
	Missing	23	40		
Is the organ donation request decoupled from conversation about futility of treatment (i.e., two different conversations in time)?	Yes	319 (37.3)	537 (62.7)	Ref.	0.015
	No	201 (44.2)	254 (55.8)	1.332 (1.057–1.679)	
	Missing	3	8		
In cases of organ donation after brain death:					
To what extent did the family understand the term ‘brain death’?^c^	Completely	289 (55.6)	231 (44.4)	33.333 (4.566–250.000)	< 0.001^b^
	Partly	27 (40.3)	40 (59.7)	18.182 (2.336–142.857)	
	Not	1 (3.6)	27 (96.4)	Ref.	
Is explicitly asked whether the family understood the term ‘brain death’?^c^	Yes	216 (57.6)	159 (42.3)	1.904 (1.381–2.625)	<0.001
	No	107 (41.6)	150 (58.3)	Ref.	
Was the physician requesting donation trained in CaD in the previous 3 years?	Yes	362 (40.8)	525 (59.2)	1.209 (0.943–1.550)	0.134
	No	138 (36.3)	242 (63.7)	Ref.	
	Missing	23	32		
Who requested donation?	ICU physician	482 (40.8)	700 (49.2)	1.721 (1.094–2.709)	0.018^b^
	Non-ICU physician	28 (28.6)	70 (71.4)	Ref.	
	Missing	13	29		
Who requested donation?	Medical specialist	412 (41.0)	592 (59.0)	1.287 (0.984–1.681)	0.064
	Resident	106 (35.1)	196 (64.9)	Ref.	
	Missing	5	11		

*CaD* Communication about Donation; *ICU* intensive care unit; *Ref.* reference group

^a^Donation intensivist assisted the physician in medical field (44.6%), procedure (53.1%), donor management (17.9%), conversation with the family (31.3%), other (7.6%); more answers possible; *n* = 224

^b^Fisher’s test (2x2 table) or Fisher-Freeman-Halton test (more than 2x2 table)

^c^This question was only applicable for donation after brain death, not for donation after circulatory death

**Fig. 2:**
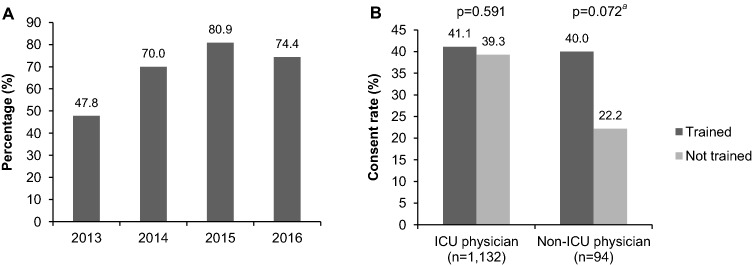
Percentage (%) of family approaches (*n* = 1322) done by a trained physician per year (2016 until June) (**a**); and family consent rates (%) of the family approaches done by trained and non-trained physicians divided in two groups: ICU physicians (*n* = 1132) and non-ICU physicians (*n* = 94)^b^ (**b**). ^a^Fisher’s exact test. ^b^From 96 physicians their function was unknown

Figure 2a shows that the percentage of family approaches done by a CaD-trained physician increased over the years. The family consent rate was 40.8% when the family was approached by a physician who had followed the CaD training in comparison with 36.3% when the family was approached by a physician who was not trained (*p* = 0.13; Table 1). Figure 2b shows the consent rates of the family approaches performed by CaD-trained compared to not-CaD-trained physicians divided in two groups: ICU physicians and non-ICU physicians. The training had more effect in the non-ICU physicians. The CaD training did not significantly affect family consent rate irrespective of the level of clinical experience (medical specialists with CaD 41.5% versus without CaD 39.3%, *p* = 0.552; residents with CaD 38.9% versus without CaD 30.8%, *p* = 0.178).

One of the questions to the physicians was: ‘which factors played a role in the decision-making process of the family?.’ The most important factors that were noted as an answer were: ‘the potential donor had previously discussed his own will with his family (60.7%),’ and ‘the personal attitude of the family toward organ donation (53.0%)’ (Table 2). Other factors are mentioned in Table 2.

**Table 2:** Predefined factors that played a role in the decision-making process of the family

Factors in decision-making process	Consent *n* = 523, *n* (%)	Objection *n* = 799, *n* (%)	Consent withdrawn^a^ *n* = 26, *n (*%)	Overall *n* = 1322, *n* (%)
The will of the deceased	318 (60.8)	485 (60.7)	11 (42.3)	803 (60.7)
The attitude of the family toward organ donation	375 (71.7)	330 (41.3)	9 (34.6)	705 (53.3)
No agreement between family members	3 (0.6)	64 (8.0)	3 (11.5)	67 (5.1)
The care and guidance in the hospital	18 (3.4)	6 (0.8)	2 (7.7)	24 (1.8)
The possibility to be present during brain diagnosis	4 (0.8)	0 (0.0)	0 (0.0)	4 (0.3)
The explanation about donation	49 (9.4)	2 (0.3)	1 (3.8)	51 (3.9)
The explanation about the content of the donation procedure	2 (0.4)	4 (0.5)	1 (3.8)	6 (0.5)
The limited time available	3 (0.6)	17 (2.1)	4 (15.4)	20 (1.5)
The duration of the procedure	9 (1.7)	49 (6.1)	17 (65.4)	58 (4.4)
Not enough space for saying farewell	3 (0.6)	19 (2.4)	3 (11.5)	22 (1.7)
Other	18 (3.4)	58 (7.3)	3 (11.5)	76^b^ (5.7)

^a^Consent withdrawn is part of the group ‘objection’

^b^Most frequently mentioned factors in category ‘other’: intact body (20.0%), family too emotional (12.0%), religious/cultural reasons (9.3%), patient had suffered enough (9.3%), other problems not related to donation (8.0%), helping other persons in need of an organ/tissue (8.0%), and difficulty accepting futility of treatment (8.0%). More than one answer could be given to this question

One of the items of the questionnaire was also whether/how the specific donation request could have been improved. A total of 159 interviewed physicians gave at least one predefined area for improvement. In total, 222 areas for improvement were suggested (Table 3). The most frequently area for improvement mentioned was more time between the conversation about futility of treatment and the donation request (34.6%). The other areas for improvement are mentioned in Table 3.

**Table 3:** Predefined areas for improvement mentioned by physicians requesting donation

Areas for improvement	*n* = 159 *n* (%)
More time between conversation about futility of treatment and donation request	55 (34.6)
More explanation about the donation procedure	26 (16.4)
Too many family members present during the donation request	26 (16.4)
More time between notification of death and donation request	20 (12.6)
Take more time for the family	16 (10.1)
Earlier contact with the donation intensivist or transplantation coordinator	14 (8.9)
Give more time for making the decision	13 (8.2)
Order of procedure: first consultation of Donor Registry, then donation request	6 (3.8)
More explanation about brain death	6 (3.8)
Other	40^a^ (25.2)

^a^Most frequently mentioned factors in category ‘other’: better coaching of the family (15.0%), donation request by ICU/trained physician (10.0%), language barrier (7.5%), earlier identification of potential donor (7.5%), busy shift (7.5%); more answers possible

## Discussion

In this nationwide study in which we analyzed 1322 consecutive organ donation requests during a 3.5-year period, we were able to identify several factors that influenced the organ donation consent rate. Surprisingly, we found that when the donation request occurred during the same conversation about futility of treatment and upcoming death (i.e., no decoupling in time), this resulted in a higher consent rate. Other independent predictors of consent to donation were understanding of the concept of ‘brain death’ and consultation of a transplant coordinator before the donation request.

### Understandable Information

Our results showed that, in case of donation after brain death, families with a good understanding of the brain death concept are more likely to consent to donation, which is consistent with the literature [6–8, 11]. It seems logical that good understanding of the brain death concept means better-informed families with less uncertainties about the organ donation process. As shown in our data, the mere fact that the requesting physician explicitly double checks with the family if they understood the concept of brain death, could be beneficial. Besides understandable information, consultation of a transplant coordinator prior to the donation request could contribute to more clarity in the donation conversations. In the Netherlands, the transplant coordinator is consulted to check medical suitability of the potential donor for donation after circulatory death or donation after brain death, for the logistic planning including timing of organ yield and organ allocation, and less frequently for supporting the donation request. Consultation of the transplant coordinator prior to the request led to higher consent rates. However, in our study, we did not specify the reason for consulting the transplant coordinator. Consultation may have led to more clarity in the conversations with the family as the requesting physician could provide more specific information regarding (suitability for) donation and approximation of the time span of logistics surrounding organ donation.

### Decoupling

In our study, we found that consent rates were higher when the donation request was not decoupled from the conversation about futility of treatment, but discussed in the same conversation. Although decoupling is often seen as an important measure to improve the consent rate, the literature shows no clear consensus on this topic. Several studies have found that decoupling is related to higher consent rates [4, 7], while other studies failed to find such a relationship [4, 12]. This may depend on the definition of ‘decoupling.’ Some state that the donation request is decoupled if it occurs after, or before, the pronouncement of death, while others if it occurs after the family accepted the futility of treatment [12]. In the Netherlands, however, the donation request always occurs before the pronouncement of death. In addition, decoupling could be created by separating the conversation in time and/or by making the request by a different person. In conclusion, the different definitions for decoupling make it difficult to adequately propose a unified method of decoupling that must be used in clinical practice. Our data suggest a refinement on this important issue.

Different authors suggest that discussing organ donation is most effective when the family accepted the loss of their loved one [5, 6, 13, 14]. When donation is requested while the family has not accepted the imminent death of their loved one, the first reaction of the family could be a rejection to a donation request. Decoupling would be the most logical thing to do in such circumstances. In other cases, the family might bring up the subject of donation themselves during the conversation about futility of treatment, which could explain why we found higher consent rates when donation was discussed in the same conversation as the futility of treatment. Indeed, several physicians noted in the questionnaires that the reason for having a donation request in the same conversation about futility of treatment was because the family was asking what would come next after hearing further treatment was futile. In such cases, decoupling would be artificial and perhaps even non-beneficial.

### Training

The literature shows that family consent rates improve if the requester is trained in communication of organ donation [8, 15–22]. In our large nationwide sample, we found that the effect of communication training seemed more pronounced in physicians that are less exposed to organ donation practices (i.e., non-ICU physicians), although this did not reach statistically significance because of a lower sample size. In the Netherlands, organ donation is mostly requested by ICU physicians. Because of the relative high mortality rate on the ICU compared to the rest of the hospital, ICU physicians are already highly specialized in end-of-life care including guidance of family members. Subsequent communication training could be less effective for such physicians because of a ceiling effect due to their experience in guiding families of dying patients. This could explain the smaller effect of the communication training in our data compared to other studies [8, 15–22].

### Strengths and Limitations

Our study presents the data of a nationwide systematic effort to evaluate all consecutive organ donation requests over a period of several years. Because of this systematic approach, we were able to obtain the data of 83% of the donation requests that occurred nationally. As our study period was 3.5 years, this resulted in a high number of donation requests we could analyze.

Although this systematic and nationwide approach minimized selection bias of cases compared to earlier reports, our study has some limitations. Most importantly, our data focused on the perspective of physicians on the family approach. Beside requester characteristics and communication processes as discussed in this study, family characteristics (e.g., religion, knowledge and attitudes about donation), deceased’s characteristics, circumstances of death, and satisfaction with hospital care could play a role in the decision-making process and were not assessed in this study [4–6, 23]. A study more focused on the family perspective would be important, but was out of the scope of this nationwide effort. In addition, as donation intensivists performed interviews with requesting physicians stationed in several hospitals in their assigned regions, interviews were not always performed directly after the family approach. This means that recall bias could have occurred. As most physicians were interviewed relatively early after their donation request, and organ donation requests occur only rarely and are accompanied with an emotional burden for the physician also, interviewed physicians remembered the cases easily when they were interviewed about the cases. Also, some bias could have been introduced by the fact that some donation intensivists performed the donation request themselves and therefore evaluated their own donation request. Lastly, we chose to include those patients that had not registered their donation wishes in the DR. The lack of donation wishes influences the family greatly. The refusal rate is highest in these cases. Although this selection introduces a bias, we chose this setup because in those patients with a registered consent, the refusal rate is low (in our sample 6.4%) and efforts to improve this low rate will not easily result in higher number of organ donors.

## Conclusion

Our data showed the complexity of successful donation requests. We showed that comprehension of the concept of brain death and consultation with transplantation coordinators could positively influence consent rates. Importantly, our data suggest that decoupling of the organ donation conversation from the conversation about futility of treatment is not always necessary and could even negatively influence consent rates.

## Electronic supplementary material

Below is the link to the electronic supplementary material.

Supplementary material 1 (DOCX 23 kb)
