# Supplementary material for: Physician Experiences with Communicating Organ Donation with the Relatives: A Dutch Nationwide Evaluation on Factors that Influence Consent Rates
Source: Neurocrit Care. 2019 Feb 14;31(2):357–64. doi: 10.1007/s12028-019-00678-8 (PMC6757095; doi:10.1007/s12028-019-00678-8)
Supplement: Supplementary file 1 — Supplementary material 1 (DOCX 23 kb) [file 12028_2019_678_MOESM1_ESM.docx]

**Supplementary Material 1**

**Evaluation form**

**Organ donation conversation**

In this Supplemental Digital Content the Dutch questionnaire is translated to English. The layout of the official questionnaire in Dutch, was not the same as this translation. Before introduction of this questionnaire, donation intensivist received additional information about its purpose, how to use it and the exact meaning of the questions. This was done nationwide in order to have a similar approach by each donation intensivist. For instance, question 10: “Did you have contact with the family before the patient died?” The purpose of this question was to evaluate if the physician who performed the organ donation request, had an earlier conversation with the family before having the conversation about futility of treatment and organ donation. For question 22: “What played an important role in the decision of the family? (more answers are possible)”. This question is about the reasons that the family gave to explain their decision about organ donation.

The questionnaire below must be used to evaluate the organ donation request with the physician who performed the request, regardless of whether consent was obtained or not. This must be done for every potential organ donor where organ donation was discussed with the relatives. This evaluation is not necessary if the deceased was registered in the Donor registry with ‘objection’.

1. Name of donation intensivist who evaluated the donation request: .........

2. Name of hospital where donation request was performed: .........

3. Region of the hospital where donation request was performed: .........

- AMS
- LB
- NY
- UT
- GR
- MS
- RD

4. Name of physician who requested for donation: .........

5. Function of physician: .........

6. Did the physician follow the Communication about Donation training in the previous 3 years?

- Yes
- No

6b. If no, did the physician follow another communication training focused on organ donation in the past 3 years?

- Yes, namely: .........
- No

7. Patient number: .........

7b. Date of death of patient: ......... (dd/mm/yyyy)

8. Was the transplantation coordinator consulted before the donation request? (for example about medical suitability)

- Yes
- No

9. What was the result of consulting the Donor registry?

- Patient leaves the decision to the relatives or a specific person
- Patient was not registered
- Patient was registered with consent
- Patient was registered with objection: stop with filling in the evaluation form.
- The Donor registry was not consulted (specify the reason below:) .........

10. Did you have contact with the family during the hospital admission before the patient died?

- Yes
- No

11. Was donation discussed during the hospital admission?

- No
- Yes, with (which family members):
  - Partner
  - Parent(s)
  - Son / daughter
  - Brother / sister
  - Combination of family members
  - Other family members
  - No family member

12. With which family members and how many family members the futility of treatment was discussed? With (which family members):

- Partner
- Parent(s)
- Son / daughter
- Combination of family members
- Other family members
- No family member

Number of persons:

- 1 -2 persons
- 3 -4 persons
- 5 -6 persons
- 7 or more persons, namely: .........

13. With which family members and how many family members did you discuss organ donation (the conversation in which the donation request was performed)? With (which family members):

- Partner
- Parent(s)
- Son / daughter
- Combination of family members
- Other family members
- No family member

Number of persons:

- 1 -2 persons
- 3 -4 persons
- 5 -6 persons
- 7 or more persons, namely: .........

14. Was the conversation about the futility of treatment decoupled from the donation request?

- Yes
- No, continue with question 16

15. Who discussed the futility of treatment and upcoming death of the patient with the family?

- The treating physician
- The (coordinating) donation intensivist
- Fellow IC
- Physician-assistant
- Other (specify function and motivate the choice for this person / professional): .........

**For donation after brain death, please fill up question 16 until 18. For donation after circulatory death continue with question 19.**

16. Did you explain the concept of brain death?

- Yes
- No, because (please explain): .........
- No, this was done by another person/professional (specify function and motivate you choice): .........

17. To what extent did the relatives understand the explanation given about the concept of brain death, in your opinion?

- Completely
- Partly
- Not

18. Did you explicitly ask whether the relatives understood the concept of brain death?

- Yes
- No, because (please explain): .........

19. To what extent are you satisfied with the way in which you conducted the donation conversation(s)?

- Unsatisfied
- Partly satisfied, partly unsatisfied
- Satisfied

Please, motivate your answer: .........

20. Which professionals, besides yourself, were involved in discussing donation with the family? (more answers are possible)

- Physician-assistant
- Nurse
- Chaplain
- Other, namely: .........

21. What was the final decision of the family?

- Consent
- Agreement (consent by family in case of consent registration in the Donor registry)
- Objection
- Objection after obtained consent
- Not applicable (because of special circumstances no decision was made. Please specify the reason below) .........

22. What played an important role in the decision of the family? (more answers are possible)

- The will of the deceased
- The attitude of the family towards organ donation
- No agreement between family members
- The care and guidance in the hospital (if necessary specify in the text box of ‘other’)
- The possibility to be present during brain diagnosis
- The explanation about donation
- The explanation about the content of the donation procedure
- The limited time available
- The duration of the procedure
- Not enough space for saying farewell
- Other, namely: .........

23. Did you get assistance of a (colleague) donation intensivist?

- Yes, he/she was physically present
- Yes, by phone
- No, specify if you get assistance from someone else (e.g. transplant coordinator), continue with question 25: .........

24. A (colleague) donation intensivist gave assistance on (….) area (more answers possible):

- Medical
- Procedural
- Donor management
- Conversation with the relatives
- Other, namely: .........

25. Summarizing conclusion on the donation conversation by (coordinating) donation intensivist:

- Donation conversation went well, no further improvement points
- Donation conversation went well, but could be improved:
  - Order of procedure: first consultation of Donor Registry, then donation request
  - More time between conversation about futility of treatment and donation request
  - More time between notification of death and donation request
  - More explanation about the donation procedure
  - More explanation about brain death
  - Take more time for the family
  - Give more time for making the decision
  - Earlier contact with the donation intensivist or transplantation coordinator
  - Too many family members present during the donation request
  - Other, namely: .........

26. Were there particularities / bottlenecks in the diagnosis of brain death?

- Not applicable
- No
- Yes, namely: .........

27. Were there particularities / bottlenecks with regard to the donor management?

- Not applicable
- No
- Yes, namely: .........

28. Were there particularities / bottlenecks with regard to the logistic planning?

- Not applicable
- No
- Yes, namely: .........

29. Date of donation conversation: ......... (dd/mm/yyyy)

30. Date of evaluation: ......... (dd/mm/yyyy)

31. How was the evaluation performed?

- Face to face
- By phone
- Per email
- Other, namely: .........

Overall comments: .........
